# High-Performance a-InGaZnO Thin-Film Transistors with Extremely Low Thermal Budget by Using a Hydrogen-Rich Al_2_O_3_ Dielectric

**DOI:** 10.1186/s11671-019-2959-1

**Published:** 2019-04-02

**Authors:** Yan Shao, Xiaohan Wu, Mei-Na Zhang, Wen-Jun Liu, Shi-Jin Ding

**Affiliations:** School of Microelectronics, Fudan University, Shanghai, 200433 People’s Republic of China

**Keywords:** Amorphous In-Ga-Zn-O, Thin-film transistor, Room temperature, Atomic layer deposition, Hydrogen-rich Al_2_O_3_

## Abstract

Electrical characteristics of amorphous In-Ga-Zn-O (a-IGZO) thin-film transistors (TFTs) are compared by using O_2_ plasma-enhanced atomic layer deposition Al_2_O_3_ dielectrics at different temperatures. High-performance a-IGZO TFTs are demonstrated successfully with an Al_2_O_3_ dielectric deposited at room temperature, which exhibit a high field-effect mobility of 19.5 cm^2^ V^− 1^ s^− 1^, a small subthreshold swing of 160 mV/dec, a low threshold voltage of 0.1 V, a large on/off current ratio of 4.5 × 10^8^, and superior negative and positive gate bias stabilities. This is attributed to the hydrogen-rich Al_2_O_3_ dielectric deposited at room temperature in comparison with higher deposition temperatures, thus efficiently passivating the interfacial states of a-IGZO/Al_2_O_3_ and the oxygen vacancies and improving conductivity of the a-IGZO channel by generating additional electrons because of enhanced hydrogen doping during sputtering of IGZO. Such an extremely low thermal budget for high-performance a-IGZO TFTs is very attractive for flexible electronic application.

## Background

Amorphous In-Ga-Zn-O (a-IGZO)-based thin film transistors (TFTs) have attracted much attention in the past decade due to their high mobility, good uniformity, high visible light transparency, and low process temperature [1–3]. These merits make it a promising candidate for the application of next-generation electronics, such as transparent display, flexible devices, or wearable electronics. In particular, for the applications of flexible electronics, TFTs are generally fabricated on low thermally stable polymer substrates. Thus, it is necessary to reduce the thermal budget of a-IGZO TFT fabrication. For this purpose, many researchers have focus on a-IGZO TFTs with room temperature fabricated gate insulators, such as sputtering [4–6], solution process [7–9], e-beam evaporation [10], and anodization [11]. However, these dielectric films often suffer from high density of traps and strong dielectric/a-IGZO interfacial scattering, thus resulting in limited field-effect mobility, a large subthreshold swing, and a small on/off current ratio [4–11].

On the other hand, atomic layer deposition (ALD) is a promising technique, which can provide high-quality films, precise control of film thickness, good uniformity over a large area, and low process temperature [12–14]. Zheng et al. [15] reported that the a-IGZO TFT with ALD SiO_2_ dielectric exhibited excellent electrical performance without the need of post-annealing. However, a high substrate temperature of 250 °C is required for the ALD of SiO_2_ films [15], which is higher than glass transition temperatures of most flexible plastic substrates. Interestingly, it is reported that ALD of Al_2_O_3_ films can be realized even at room temperature (RT) [16, 17]; meanwhile, the Al_2_O_3_ film deposited at RT contains a large amount of hydrogen (H) impurities [17]. However, to the best of our knowledge, the abovementioned H-rich Al_2_O_3_ film has never been utilized as a gate insulator in a-IGZO TFT. Therefore, it is desirable to explore the a-IGZO TFT with a RT ALD Al_2_O_3_ gate insulator.

In this letter, high-performance a-IGZO TFT was successfully fabricated with a room temperature deposited Al_2_O_3_ gate dielectric. By comparing the characteristics of the a-IGZO TFTs with various Al_2_O_3_ gate insulators deposited at different temperatures, the underlying mechanism was addressed.

## Methods

Highly doped p-type silicon wafers (< 0.0015 Ω cm) were cleaned by standard RCA processes and served as gate electrodes. Forty-nanometer Al_2_O_3_ films were deposited in a commercial ALD system (Picsun Ltd.) using trimethylaluminum (TMA) and O_2_ plasma as a precursor and reactant, respectively. One growth cycle consisted of 0.1 s TMA pulse, 10 s N_2_ purge, 8 s O_2_ plasma pulse, and 10 s N_2_ purge. The TMA was maintained at 18 °C for a stable vapor pressure and dose, and the O_2_ gas flow rate was fixed at 150 sccm with a plasma generator power of 2500 W. Subsequently, 40-nm a-IGZO films were deposited by RF sputtering using an IGZO ceramic target with an atomic ratio of In:Ga:Zn:O = 1:1:1:4. During sputtering, working pressure and Ar and O_2_ gas flow rates were fixed at 0.88 Pa and 48 and 2 sccm, respectively. The active region was formed by photolithography and wet etching. After that, source/drain electrodes of 30-nm Ti/70-nm Au bilayers were prepared by electron beam evaporation and a lift-off method. No further annealing processes were applied on these devices.

The electrical properties of a-IGZO TFTs were characterized using a semiconductor device analyzer (Agilent Tech B1500A) in a dark box at room temperature. The device stabilities were measured under positive and negative gate bias stresses, respectively. The depth profiles of elements and chemical composition were measured by secondary ion mass spectrometry (SIMS) and X-ray photoelectron spectroscopy (XPS), respectively.

## Results and Discussion

**Fig. 1:**
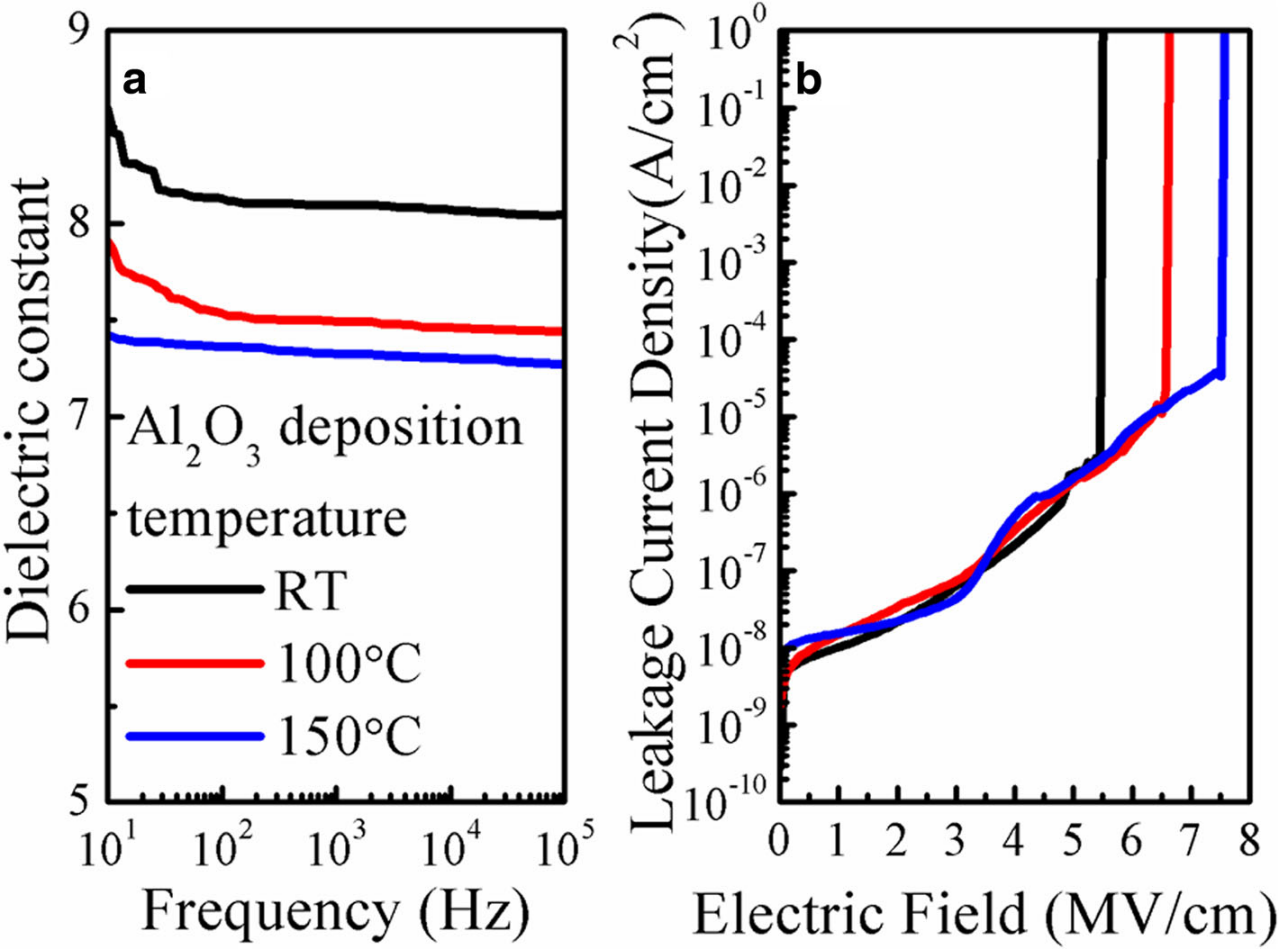
Electrical properties of Al_2_O_3_ films deposited at different temperatures. **a** Dielectric constant versus frequency. **b** Leakage current density versus electric field

Figure 1a compares the dielectric constants of the Al_2_O_3_ films deposited at different temperatures as a function of frequency (i.e., from 10 Hz to 10^5^ Hz). As the deposition temperature increases from 100 to 150 °C, the film shows a gradual decrease in dielectric constant. A similar trend was also reported in previous literatures for the deposition temperature changing from RT to 150 °C [18, 19]. This is because the RT Al_2_O_3_ film contains the highest concentration of hydrogen (H) in the form of OH groups. Thus, the corresponding dielectric constant is enhanced due to a rotation of more OH groups in an electric field [20]. In terms of the measurement frequency of 10 Hz, the extracted dielectric constants for the RT, 100 °C, and 150 °C Al_2_O_3_ films are equal to 8.6, 7.9, and 7.4, respectively, which are used for the extraction of the field-effect mobility (*μ*_FE_) and interfacial trap density (*D*_it_) of the fabricated TFT device. Figure 1b shows the leakage current characteristics of different Al_2_O_3_ films. It is found that the RT Al_2_O_3_ film exhibits a small leakage current density of 2.38 × 10^− 8^ A/cm^2^ at 2 MV/cm and a breakdown electric field of 5.3 MV/cm. In addition, the breakdown electric field increases gradually with increasing deposition temperature from 100 to 150 °C.

**Fig. 2:**
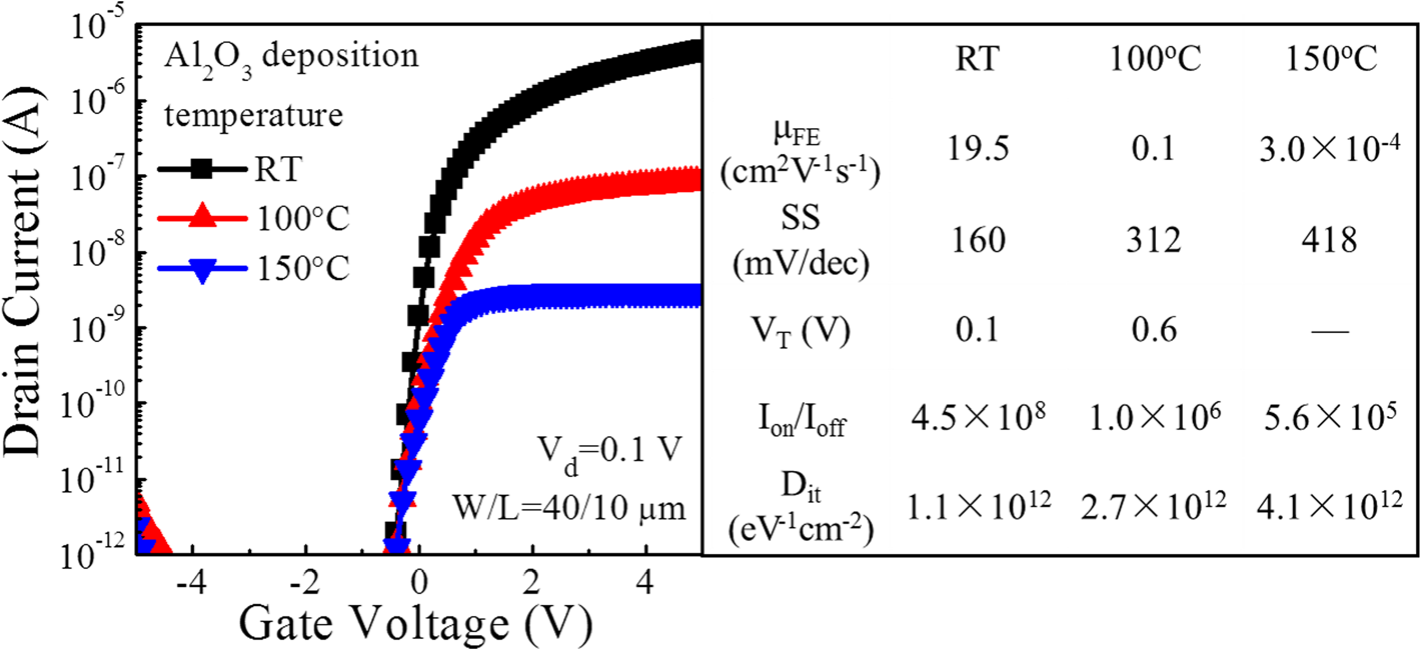
Transfer curves of the a-IGZO TFTs with ALD Al_2_O_3_ gate insulators deposited at different temperatures together with the extracted device parameters

Figure 2 shows the typical transfer curves of the a-IGZO TFTs with different Al_2_O_3_ gate insulators. The RT Al_2_O_3_ TFT exhibits the best performance, such as high *μ*_FE_ of 19.5 cm^2^ V^− 1^ s^− 1^, a small subthreshold swing (SS) of 160 mV/dec, a small threshold voltage (*V*_T_) of 0.1 V, and a large on/off current ratio (*I*_on/off_) of 4.5 × 10^8^. However, the a-IGZO TFTs with Al_2_O_3_ gate insulators deposited at both 100 and 150 °C show a much poorer performance, i.e., reduced on-currents (10^− 7^ and 3 × 10^− 9^ A) and degraded SS. The *D*_it_ at the interface of Al_2_O_3_/a-IGZO can be calculated based on the following equation [21]:

1 $$ {D}_{\mathrm{it}}=\left(\frac{\mathrm{SS}\times \lg e}{kT/q}-1\right)\frac{C_{ox}}{q^2} $$

where *e*, *k*, *T*, and *q* represent the Euler’s number, Boltzmann constant, absolute temperature, and unit electron charge, respectively. *C*_ox_ is the gate dielectric capacitance per unit area. For the RT Al_2_O_3_ TFT, the *D*_it_ is equal to 1.1 × 10^12^ eV^− 1^ cm^− 2^, which is over one or two times lower than those for the TFTs with the Al_2_O_3_ gate insulators deposited at 100 and 150 °C.

**Fig. 3:**
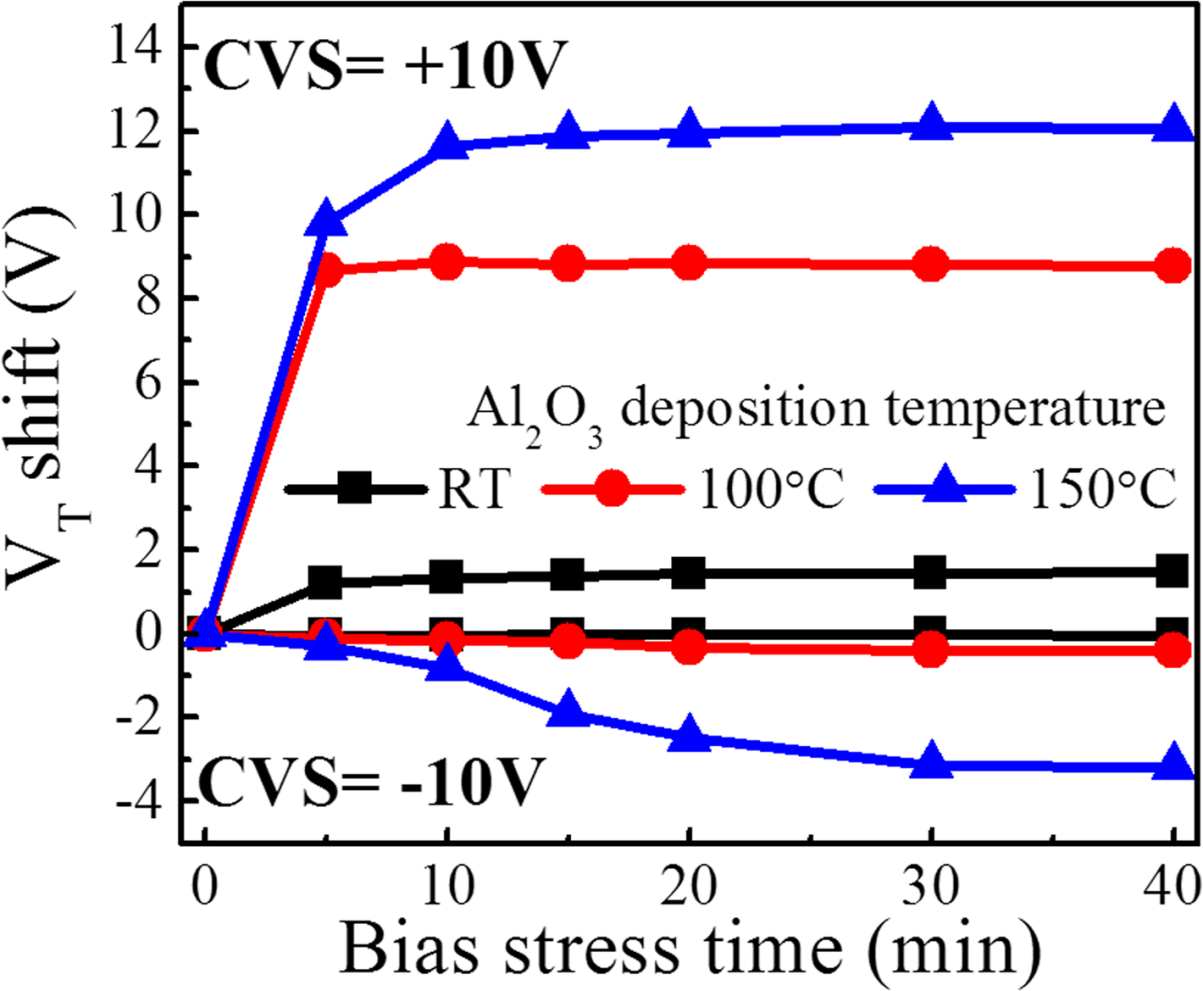
*V*_T_ shift as a function of bias stress time under NGBS = − 10 V and PGBS = 10 V for the TFTs with Al_2_O_3_ insulators deposited at different temperatures

**Fig. 4:**
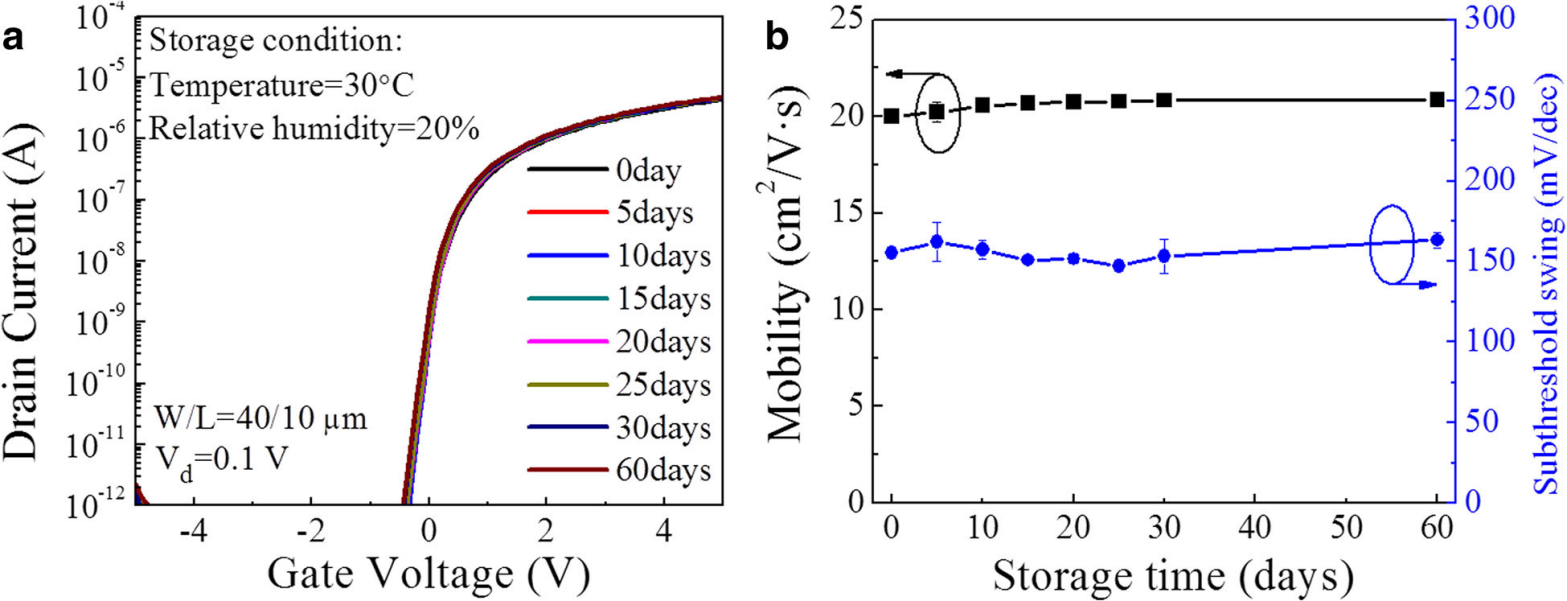
Time-dependent stability of RT Al_2_O_3_ TFT after being kept in a cabinet (20% RH) at 30 °C. **a** Transfer curves. **b** Mobility and subthreshold swing

The gate bias stabilities of the devices were further measured by applying negative and positive voltages. Figure 3 shows the *V*_T_ shift as a function of bias stress time for different TFTs. In terms of negative gate bias stress (NGBS), the RT Al_2_O_3_ TFT exhibits a negligible *V*_T_ shift of − 0.04 V after being stressed at − 10 V for 40 min. However, higher-temperature Al_2_O_3_ gate insulators generate larger *V*_T_ shifts especially for 150 °C. Such a high NGBS stability for RT Al_2_O_3_ should be attributed to a low concentration of oxygen vacancies (*V*_O_) in the a-IGZO channel [22]. With respect to positive gate bias stress (PGBS), the RT Al_2_O_3_ TFT shows a *V*_T_ shift of 1.47 V, which is much smaller than those (8.8 V and 12.1 V) for the 100 and 150 °C Al_2_O_3_ TFTs. Moreover, the influence of storage time on the device performance was investigated, as shown in Fig. 4. Although no passivation layer is covered on the back channel, the device still maintains an excellent performance after being kept in a cabinet (20% RH) for 60 days at 30 °C; meanwhile, no significant variations in *μ*_FE_ and SS are observed. This indicates the RT Al_2_O_3_ TFTs without any passivation layer have good storage-time-dependent stability in the current ambience.

Table 1 compares the performance of our RT Al_2_O_3_ TFT with other reports. It is found that our device exhibits a zero-near *V*_T_, smaller SS, and larger *I*_on/off_ in the case of comparable mobility [4, 23]. Although using a Ta_2_O_5_ gate insulator can obtain higher mobility of 61.5 cm^2^ V^− 1^ s^− 1^, both SS and *I*_on/off_ deteriorate remarkably [10]. In a word, our RT Al_2_O_3_ TFT possesses a superior comprehensive performance in comparison with the 100 and 150 °C Al_2_O_3_ TFTs. Since all processing steps are identical except the deposition step of Al_2_O_3_, such significant differences in electrical performance should originate from the Al_2_O_3_ gate insulators.

**Table 1:** Comparison of the electrical parameters of our RT Al_2_O_3_ a-IGZO TFT and other a-IGZO TFTs fabricated at low temperatures (*T*_max_ denotes the maximum process temperature)

Insulator	*T*_max_ (°C)	*μ*_EF_ (cm^2^ V^− 1^ s^− 1^)	SS (V/dec)	*V*_T_ (V)	*I*_on_/_off_	Ref.
Al_2_O_3_	RT	19.5	0.16	0.1	4.5 × 10^8^	This work
Al_2_O_3_	RT	7.5	0.44	–	3.1 × 10^8^	[31]
Al_2_O_3_	60	5.9	0.26	2.48	10^8^	[9]
SiO_2_	RT	18.5	0.27	1.5	10^7^	[4]
SiO_2_	RT	15.8	0.66	−0.42	4.4 × 10^5^	[23]
SiO_2_	90	11	0.4	0.44	10^5^	[32]
Ta_2_O_5_	RT	61.5	0.61	0.25	10^5^	[10]

**Fig. 5:**
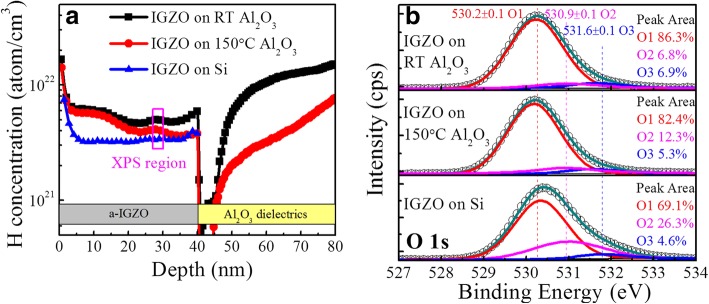
**a** SIMS profiles of hydrogen concentration in Al_2_O_3_ deposited at RT and 150 °C. **b** High-resolution O1s XPS spectra of the IGZO channel deposited on RT Al_2_O_3_, 150 °C Al_2_O_3_, and bare Si

To understand the underlying mechanism, the depth profiles of the elements in the a-IGZO/Al_2_O_3_ stacked films were analyzed by SMIS. Figure 5a shows the dependence of H concentration on depth in the stacks of IGZO/Al_2_O_3_, where the Al_2_O_3_ films were deposited at RT and 150 °C, respectively. For comparison, an IGZO film deposited on a bare Si substrate was also analyzed. The IGZO film deposited on bare Si contains an H concentration of ~ 3 × 10^21^ cm^− 3^, which originates from the residual gas in sputtering system and absorbed H_2_/H_2_O molecules on the Si surface. Both IGZO films deposited on the Al_2_O_3_ films contain higher H concentrations than that on the bare Si substrate. This indicates that the increased H concentrations should come from the release of H impurities in the underlying Al_2_O_3_ films during sputtering of IGZO. Moreover, it is observed that the H concentration in the IGZO film atop the RT Al_2_O_3_ film is higher than that on the 150 °C one in the interface-near region, which can provide more efficient passivation of interfacial states. This thus improves the SS and PGBS stability of the RT Al_2_O_3_ TFT by reducing interfacial carrier trapping. Additionally, the O 1s XPS spectra of the a-IGZO films near the interface of IGZO/Al_2_O_3_ were analyzed, as shown in Fig. 5b. The fitted peaks are located at 530.2 ± 0.1 eV, 530.9 ± 0.1 eV, and 531.6 ± 0.1 eV, corresponding to O^2−^ ions bound with metal (O1), *V*_O_ (O2), and OH groups (O3), respectively [13, 24]. The percentage of O2 is 26.3% in the a-IGZO layer atop the bare Si; however, it decreases to 12.3% and 6.8% for the 150 °C and RT Al_2_O_3_ underlying films, respectively. This indicates that more *V*_O_ in the IGZO channel can be effectively passivated by additional H impurities originating from the underlying Al_2_O_3_ films, especially for the RT Al_2_O_3_ film with a higher H concentration. It is reported that when *V*_O_ and H both are present in the a-IGZO film, they can combine to form a stable state in which H is trapped at *V*_O_ (*V*_O_H), and the resulting *V*_O_H is a shallow-level donor [25–27]. Thus, enhanced H doping into the IGZO channel atop the RT Al_2_O_3_ improves the channel conductivity by providing additional electrons. Furthermore, the small *V*_T_ shift under the NGBS for the RT Al_2_O_3_ TFT can also be attributed to the effective H passivation of *V*_O_ [28]. As reported in literatures, the instability of TFT under NGBS originates from ionization of neutral *V*_O_ (*V*_O_ → *V*_O_^2+^+2e^−^) [17, 29]. Moreover, the O3 percentage of the a-IGZO film on the RT Al_2_O_3_ is 6.9%, which is higher than those on the 150 °C Al_2_O_3_ (5.3%) and the bare Si (4.6%), respectively. The OH group could originate from the reaction O^2−^ + H → OH^−^ + e^−^ during deposition of IGZO films [30]. Thus, the enhanced H doping into the IGZO channel atop the RT Al_2_O_3_ film generates more OH groups and also contributes to improve the channel conductivity.

## Conclusions

A high-performance a-IGZO TFT was fabricated successfully under the extremely low thermal budget of RT using an H-rich Al_2_O_3_ gate dielectric prepared by O_2_ plasma-enhanced ALD. This is ascribed to the fact that the Al_2_O_3_ dielectric deposited at RT contains more hydrogen impurities than those deposited at higher temperatures. Thus, the released H impurities during sputtering of IGZO generated more electrons, and efficiently passivated the interfacial states of a-IGZO/Al_2_O_3_ and the *V*_O_ in the a-IGZO channel.

## Abbreviations

a-IGZO: Amorphous In-Ga-Zn-O
ALD: Atomic layer deposition
*D*_it_: Interfacial trap density
H: Hydrogen
*I*_on/off_: On/off current ratio
NGBS: Negative gate bias stress
PGBS: Positive gate bias stress
RT: Room temperature
SIMS: Secondary ion mass spectrometry
SS: Subthreshold swing
TFT: Thin-film transistor
*V*_O_: Oxygen vacancy
*V*_O_H: Hydrogen trapped at oxygen vacancy
*V*_T_: Threshold voltage
XPS: X-ray photoelectron spectroscopy
*μ*_FE_: Field-effect mobility
